# Bayesian network meta-analysis of the impact of exercise therapy on blood glucose in type 2 diabetes patients

**DOI:** 10.3389/fendo.2025.1658739

**Published:** 2025-11-03

**Authors:** Hairong Liu, Wenli Wang, Jing Sun

**Affiliations:** ^1^ Faculty for Physical Education, Shanghai International Studies University, Shanghai, China; ^2^ Faculty for Basic Education, Shanghai Institute of Visual Arts, Shanghai, China

**Keywords:** type 2 diabetes, exercise therapy, blood glucose, network meta-analysis, randomized controlled trial

## Abstract

**Objective:**

To systematically evaluate the effects of exercise therapy on glycemic control in patients with type 2 diabetes mellitus (T2DM) and compare the efficacy of different exercise regimens.

**Methods:**

Randomized controlled trials (RCTs) investigating exercise interventions in T2DM were identified through systematic searches of PubMed, The Cochrane Library, Web of Science, Embase, China National Knowledge Infrastructure (CNKI), VIP, Wanfang Database, and China Biology Medicine (CBM) from inception to September 2024. Methodological quality was assessed using the Physiotherapy Evidence Database (PEDro) scale. Data were analyzed using RevMan 5.4, Stata 15.1, R 4.0.5, and JAGS 4.3.0 for Bayesian network meta-analysis.

**Results:**

A total of 22 studies involving 1448 participants were included. All studies were RCTs, with PEDro scores ranging from 6 to 8, with an average score of 6.41, indicating overall high quality. Exercise therapy significantly improved fasting blood glucose (SMD = -0.58, 95%CI: [-0.77, -0.39], *P* < 0.00001), 2-hour postprandial blood glucose (SMD = -0.51, 95%CI: [-0.73, -0.30], *P* < 0.00001), and glycated hemoglobin (MD = -0.66, 95%CI: [-0.78, -0.54], *P* < 0.00001). Aerobic exercise combined with resistance exercise for 14–24 weeks yielded the best improvement in fasting blood glucose (SMD = -2.106, 95%CI: [-3.042, -1.176]), 2-hour postprandial blood glucose (SMD = -3.177, 95%CI: [-5.623, -0.791]), and glycated hemoglobin (MD = -1.382, 95%CI: [-2.050, -0.747]).

**Conclusion:**

Exercise therapy is an effective non-pharmacological approach for improving glycemic control in T2DM. Based on current evidence, combined aerobic and resistance exercise for 14–48 weeks is recommended as the optimal strategy for reducing fasting blood glucose, 2-hour postprandial blood glucose, and glycated hemoglobin levels in this population.

**Systematic Review Registration:**

www.crd.york.ac.uk, identifier CRD420251069226.

## 1 Introduction

Diabetes is recognized as the third leading chronic non-communicable disease globally, following cardiovascular diseases and cancer. Type 2 diabetes mellitus (T2DM) comprises over 90% of all diabetes cases (1), with a prevalence rate of 10.4% among adults in China, and exceeding 20% among individuals aged 60 and older (2). T2DM is primarily characterized by hyperglycemia. Chronic hyperglycemia can result in metabolic disturbances, significantly increasing the risk of cardiovascular diseases and other complications (3), thereby posing serious threats to health and even life (4). Projections indicate that by 2045, the number of individuals with diabetes in China is expected to increase to 174 million, ranking first in the world (5).

Exercise therapy is a cornerstone treatment for T2DM and should be consistently integrated into all aspects of its management. Research has shown that insufficient physical activity and sedentary behavior significantly elevate the risk of developing T2DM (6). Exercise improves insulin sensitivity and addresses metabolic disturbances in T2DM patients, aiding in blood sugar control, reducing mortality rates, and preventing the onset of cardiovascular diseases (7). Meta-analyses have demonstrated that exercise significantly enhances glucose and lipid metabolism in individuals with prediabetes, leading to reductions in blood sugar levels (8). Prospective studies indicate that regular physical activity enables 46.9% of T2DM patients to achieve normal blood sugar levels without relying on hypoglycemic medications (9). Longitudinal studies reveal that following exercise interventions, β-cell functionality significantly improves in T2DM patients, accompanied by increased insulin secretion and reduced blood sugar levels (10).

Previous studies have demonstrated that both aerobic exercise and resistance training significantly improve glycosylated hemoglobin levels and contribute to weight loss (11). Additionally, high-intensity interval training, along with a combination of aerobic and resistance exercises, has been particularly effective in enhancing blood glucose and lipid profiles (12–14). Exercise therapy has been shown to significantly enhance blood glucose metrics in individuals with Type 2 Diabetes Mellitus (T2DM) (15–17). Moreover, we note that previous research has paid little attention to the effects of different levels of exercise elements (e.g., type, duration, intensity, frequency, and period) on glycemic control, and has seldom addressed the complex interactions among various combinations of these elements. To bridge this gap, the present study employs an integrated approach combining traditional meta-analysis and network meta-analysis. The conventional meta-analysis was conducted to evaluate the overall efficacy of exercise therapy on fasting blood glucose, 2-hour postprandial blood glucose, and glycated hemoglobin (HbA1c) in patients with type 2 diabetes mellitus (T2DM), and to identify key exercise-related factors. Building on these findings, we further performed a Bayesian network meta-analysis to directly and indirectly compare multiple intervention strategies formed by different combinations of exercise elements, and to probabilistically rank their relative effectiveness. This approach allows us to provide a hierarchical recommendation of preferred exercise type and period combinations. Ultimately, this study aims to offer an individualized, precise, and clinically applicable exercise prescription for improving glycemic dysregulation in T2DM, thereby contributing evidence-based support for clinical rehabilitation and care.

## 2 Research methods

This research adheres to the international guidelines for conducting meta-analyses (18) in the selection and application of research methods, and is registered with PROSPERO under the number: CRD420251069226. Institution: National Institute for Health and Care Research (NIHR). Registration platform: www.crd.york.ac.uk.

### 2.1 Literature search strategy

Two researchers performed literature searches across eight databases: PubMed, The Cochrane Library, Web of Science, Embase, China National Knowledge Infrastructure (CNKI), Weipu, Wanfang, and the China Biomedical Literature Database. They complemented this with reference tracing, with the search timeframe extending from the establishment of these databases to September 2024, focusing on randomized controlled trials evaluating exercise therapy for Type 2 Diabetes Mellitus. The search strategy employed a combination of subject terms and free-text keywords. The Chinese search terms included: Type 2 Diabetes Mellitus, diabetes, non-insulin-dependent diabetes, exercise therapy, physical exercise, aerobic exercise, resistance training, and randomized controlled trials. The English search terms included: Diabetes Mellitus, Type 2; Diabetes Mellitus, Noninsulin-Dependent; Diabetes Mellitus, Maturity-Onset; Type 2 Diabetes; Exercise Therapy; Remedial Exercise; Rehabilitation Exercise; Aerobic Exercise; Resistance Training; Randomized Controlled Trial; and Randomized. As an example, the search strategy for PubMed was as follows:

#1 “diabetes mellitus, type 2” [MeSH Terms] OR “Diabetes Mellitus, Noninsulin-Dependent” [Title/Abstract] OR “Diabetes Mellitus, Ketosis-Resistant” [Title/Abstract] OR “Diabetes Mellitus, Non Insulin Dependent” [Title/Abstract] OR “Diabetes Mellitus, Stable” [Title/Abstract] OR “Diabetes Mellitus, Type II” [Title/Abstract] OR “Diabetes Mellitus, Maturity-Onset” [Title/Abstract] OR “Diabetes Mellitus, Slow-Onset” [Title/Abstract] OR “Noninsulin-Dependent Diabetes Mellitus” [Title/Abstract] OR “Maturity-Onset Diabetes” [Title/Abstract] OR “Type 2 Diabetes” [Title/Abstract] OR “Diabetes Mellitus, Adult-Onset” [Title/Abstract].

#2 “exercise therapy” [MeSH Terms] OR “Remedial Exercise” [Title/Abstract] OR “Rehabilitation Exercise” [Title/Abstract] OR “Exercise” [Title/Abstract] OR “Physical Activity” [Title/Abstract] OR “Exercise, Physical” [Title/Abstract] OR “Aerobic Exercise” [Title/Abstract] OR “Resistance Training” [Title/Abstract] OR “Strength Training” [Title/Abstract].

#3 “randomized controlled trial” [Publication Type] OR randomized [Title/Abstract].

#4 #1 AND #2 AND #3.

### 2.2 Inclusion and exclusion criteria for literature

#### 2.2.1 Inclusion criteria

The inclusion criteria for the selected literature were established according to the PICOS principles of evidence-based medicine.(1) Participants: Individuals clinically diagnosed with Type 2 Diabetes Mellitus, regardless of gender, age, race, or nationality, who do not have other comorbidities, mental disorders, severe cognitive impairments, or significant organic diseases; (2) Interventions: Exercise therapy, encompassing interval aerobic exercise, continuous aerobic exercise, resistance training, and a combination of aerobic and resistance exercises; (3) Control Groups: In traditional meta-analyses, the control group is the usual care group. In network meta-analyses, studies may also be included if they lack a usual care group but contain multiple (≥2) exercise intervention groups; (4) Outcome Measures: Fasting blood glucose (FBG), 2-hour postprandial blood glucose (2hPG), and glycosylated hemoglobin (HbA1c); (5) Study Types: Randomized controlled trials (RCTs).

#### 2.2.2 Exclusion criteria

(1) Animal studies; (2) Review articles and conference abstracts; (3) Studies involving combined interventions that integrate additional therapeutic methods along with exercise interventions; (4) Studies for which data could not be extracted, and where original data were not provided even after contacting the authors; (5) Studies with low methodological quality.

### 2.3 Literature screening, data extraction, and quality assessment

#### 2.3.1 Literature screening and data extraction

The articles retrieved from each database were imported into Endnote software for deduplication. Two researchers independently screened the literature based on the inclusion and exclusion criteria, extracted relevant information, and cross-checked their findings. In instances of disagreement, a third researcher participated in the discussion to decide on inclusion. A standardized data extraction form was utilized to gather information, including the first author’s name, publication year, country, sample size, age, exercise duration, frequency, time, intensity, intervention measures, and outcome indicators.

#### 2.3.2 Quality assessment

The methodological quality of the randomized controlled trials was evaluated using the Physiotherapy Evidence Database (PEDro) scale. This evaluation was carried out independently by two researchers, and in cases of disagreement, a third researcher was involved to discuss and resolve the issues. The scale includes 11 items, and scoring is based on items 2 through 11. Each item that meets the criteria scores 1 point; items that do not meet the criteria or are unclear score 0 points, yielding a maximum total score of 10 points. Scores of 9–10 denote high-quality research, scores of 6–8 indicate moderate-quality research, scores of 4–5 reflect average-quality research, and scores below 4 indicate low-quality research.

### 2.4 Data processing

The traditional meta-analysis was performed using RevMan 5.4 software to conduct statistical analyses. Heterogeneity was evaluated using *P*-values and *I*². If the studies exhibited statistical heterogeneity (*I*² ≥ 50%, *P* < 0.10), a random effects model was utilized; otherwise, a fixed effects model was applied. Continuous data measured with the same tools were expressed as weighted mean differences (MD) along with their 95%CI; otherwise, the standardized mean differences (SMD) and their 95% CI were reported. A meta-analysis and subgroup analysis of all outcome indicators from the included literature were conducted using RevMan 5.4.

For the network meta-analysis, all statistical computations and model diagnostics were conducted using R (version 4.0.5) and JAGS (version 4.3.0). Network graphs and funnel plots were generated with Stata (version 15.1). In the network graph, each node represents an individual intervention, with the size of the node proportional to the sample size allocated to that intervention. Edges between nodes indicate the presence of direct comparative evidence, and the thickness of each edge corresponds to the number of available direct-comparison studies. This visualization clearly illustrates the architecture of the evidence network formed by the included interventions. All outcome indicators in this study were continuous variables, utilizing either the weighted mean difference (MD) or standardized mean difference (SMD) as the effect size, with 95%CI calculated (a CI that does not include 0 indicates a statistically significant difference). A Bayesian Markov Chain Monte Carlo (MCMC) model was utilized to compare the different exercise programs. Initially, four chains were established for the simulation, with an iteration step length of 1, totaling 50,000 iterations, of which the first 20,000 iterations were designated for burn-in to mitigate the impact of initial values (19). Heterogeneity was quantified using the *I*² statistic. Node analysis was employed to evaluate consistency. If *P* > 0.05, it indicated that there was no significant difference between direct and indirect comparisons, and a consistency model was chosen; otherwise, an inconsistency model was applied. When closed-loop structures are present among the interventions, both global and local inconsistency tests must be conducted to assess the consistency of each loop using inconsistency factors and their 95%CI; a smaller inconsistency factor with a 95%CI that includes 0 suggests good loop consistency. The potential scale reduction factor (PSRF) was used to assess the iterative effects of variance both between and within model chains. If the PSRF approaches or equals 1, it indicates good model convergence and stability, suggesting that the results from the consistency model analysis are highly trustworthy; otherwise, further expansion of the model calculations may be necessary. The area under the surface of the cumulative ranking (SUCRA) was utilized to indicate the effectiveness ranking of each intervention; higher values signify better intervention effects (20).

## 3 Results

### 3.1 Literature search results

**Figure 1:**
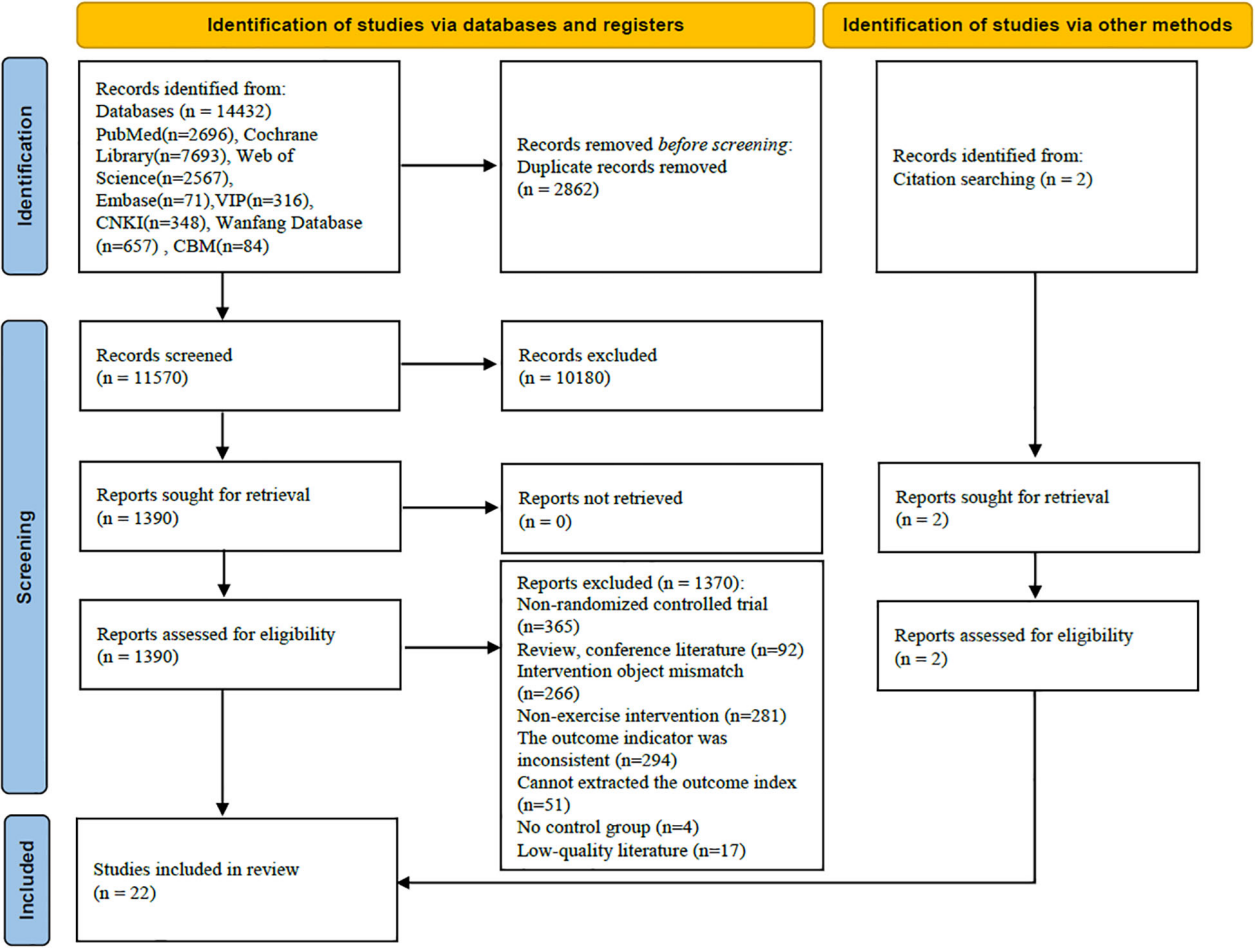
Flowchart of literature screening.

A total of 14,432 relevant articles were identified, with 22 randomized controlled trials (RCTs) included in the analysis. The process and results of the literature screening are illustrated in **Figure 1**.

### 3.2 Basic characteristics of included studies

This study incorporated 22 articles encompassing a total of 1,448 patients. The traditional meta-analysis was limited to articles that utilized a conventional treatment group as the control, resulting in a total of 18 studies (21–38). The network meta-analysis expanded upon the literature included in the traditional meta-analysis by adding four studies (39–42) that did not have a conventional treatment group and featured multiple exercise intervention groups, culminating in a total of 22 articles. The study participants were patients clinically diagnosed with type 2 diabetes, and the interventions included intermittent aerobic exercise, continuous aerobic exercise, resistance training, and a combination of aerobic and resistance training. The fundamental characteristics of the included studies are detailed in **Table 1**.

**Table 1:** Basic characteristics of the included literature.

Study	Nation	Sample size T/C	Age (years) T/C	Intervention measure					Outcome Indicator
				Form of intervention	Period (week)	Frequency (times/week)	Duration (min/time)	Intensity	
Church 2010 (21)	United States of America	73/72/76/41	56.9 ± 8.7/53.7 ± 9.1/ 55.4 ± 8.3/ 58.6 ± 8.2	T1: Resistance exercise T2: Continuous aerobic exercise T3: Combined aerobic and resistance exercise C: Conventional treatment	36	3	60	T1: medium intensity; T2:50-80%VO2max; T3: medium intensity+ 50-80%VO2max	③
Jorge 2011 (22)	Brazil	12/12/12/12	52.09 ± 8.71/ 54.10 ± 8.94/ 57.90 ± 9.82/ 53.42 ± 9.82	T1: Continuous aerobic exercise T2: Resistance exercise T3: Combined aerobic and resistance exercise C: Conventional treatment	12	3	60	—	①②③
Karstoft 2013 (23)	Denmark	12/12/8	57. 5 ± 2.4/ 60. 8 ± 2.2/ 57. 1 ± 3.0	T1: Intermittent aerobic exercise T2: Continuous aerobic exercise C: Conventional treatment	16	5	60	T1:>70%VO2max +<70%VO2max; T2:55%VO2max	①②③
Oliveira 2012 (24)	Brazil	11/10/10/12	52.09 ± 8.71/ 54.10 ± 8.94/ 57.90 ± 9.82/ 53.42 ± 9.82	T1: Continuous aerobic exercise T2: Resistance exercise T3: Combined aerobic and resistance exercise C: Conventional treatment	12	3	60	T1: Heart rate corresponding to lactate threshold; T2:50%1RM; T3: Heart rate corresponding to lactate threshold+ 50%1RM	①②③
Park 2015 (25)	South Korea	24/13	71.2 ± 3.9/69.6 ± 3.6	T: Combined aerobic and resistance exercise C: Conventional treatment	12	3	60	RPE:9-14+ 45-75%1RM	③
Shenoy 2009 (26)	India	10/10/10	49.6 ± 5.2/ 52.2 ± 9.3/ 58.4 ± 1.8	T1: Resistance exercise T2: Continuous aerobic exercise C: Conventional treatment	16	T1:2; T2:3	30	T1:60-100%1RM T2: —	①③
Tan 2012 (27)	China	15/10	65.9 ± 4.2/ 64.8 ± 6.8	T: Combined aerobic and resistance exercise C: Conventional treatment	24	3	40	55-70%HRmax+50-70%1RM	①②③
Winding 2018 (28)	Denmark	13/12/7	54 ± 6/ 58 ± 8/ 57 ± 7	T1: Intermittent aerobic exercise T2: Continuous aerobic exercise C: Conventional treatment	11	3	T1:20; T2:40	T1:95% peak volume+Intermittent20%peak volume;T2:50%peak volume	①②③
Yavari 2012 (29)	Iranian	15/15/15/15	48.2 ± 9.2/ 51.5 ± 6.3/ 50.9 ± 9.8/51.5 ± 8.5	T1: Continuous aerobic exercise T2: Resistance exercise T3: Combined aerobic and resistance exercise C: Conventional treatment	48	3	20-60	T1:60-75%HRmax; T2:60-80%1RM; T3:60-75%HRmax+60-80%1RM	①②③
Huilan Cheng 2013 (30)	China	30/30	69.5 ± 5.7	T: Resistance exercise C: Conventional treatment	9	3	30	45-55%VO2max	①②③
Jiangping Hu 2019 (31)	China	15/15/15/15	57.14 ± 4.19	T1: Continuous aerobic exercise T2: Continuous aerobic exercise T3: Continuous aerobic exercise C: Conventional treatment	12	3	60	T1:60%HRmax; T2:75-80%HRmax; T3:60%HRmax+75-80%HRmax+60%HRmax	①②③
Tongxin Li 2014 (32)	China	23/25/21	56.43 ± 7.01/55.63 ± 6.78/56.28 ± 6.38	T1: Continuous aerobic exercise T2: Resistance exercise C: Conventional treatment	12	4	T1: 20-50	T1:50-60%VO2max; T2: Target heart rate (220-age)×50%-60%	①②③
Yishu Wang 2016 (33)	China	20/20	66.5 ± 6.3/ 69.2 ± 7.8	T: Resistance exercise C: Conventional treatment	12	3	30	—	①②③
Yuxin Xu 2019 (34)	China	40/44/36	65.41 ± 5.01/ 65.46 ± 4 98/ 66.32 ± 5.35	T1: Continuous aerobic exercise T2: Combined aerobic and resistance exercise C: Conventional treatment	24	5	30	T1:50-65%HRmax; T2:50-65%HRmax% 70 -80%1RM	①③
Qing Meng 2018 (35)	China	40/40/40		T1: Continuous aerobic exercise T2: Combined aerobic and resistance exercise C: Conventional treatment	12	3	60	T1:50-80HRmax;T2:Borg12~13	①③
Qian Liu 2021 (36)	China	50/50	8 people<55 years old, 42 people 55~75 years old/10 people<55 years old, 40 people 55~75 years old	T: Combined aerobic and resistance exercise C: Conventional treatment	12	Aerobic 5 Resistant 2	30	Medium-low intensity	①②③
Qi Gu 2021 (37)	China	23/23/21	63.5 ± 6.3/ 64.2 ± 6.7/ 64.5 ± 7.6	T1: Combined aerobic and resistance exercise T2: Combined aerobic and resistance exercise C: Conventional treatment	12	T1:2; T2:3	T1:60; T2:50	T1:55-60% HRmax+70%1RM; T2:55-60% HRmax+40%1RM	①②③
Yanping Wu 2022 (38)	China	23/21/18	54 ± 7/ 57 ± 8/ 56 ± 6	T1: Intermittent aerobic exercise T2: Continuous aerobic exercise C: Conventional treatment	12	3	T1:35; T2:60	T1:90%Hrmax+Intermittent T2:70%Hrmax	①②③
Ng 2010 (39)	Singapore	30/30	57 ± 7/ 59 ± 7	T1: Resistance exercise T2: Continuous aerobic exercise	8	2-3	T1:50; T2:50	T1:65-70%1RM; T2:65-70%HRmax	①③
Terada 2013 (40)	Canada	8/7	62 ± 3.0/ 63 ± 5.0	T1: Intermittent aerobic exercise T2: Continuous aerobic exercise	12	5	30-60	T1:100%Oxygen consumption reserve+Intermittent 20%Oxygen consumption reserve; T2:40%Oxygen consumption reserve	①③
Xiujun Zhao 2019 (41)	China	21/20	17.76 ± 2.54/ 17.20 ± 2.19	T1: Continuous aerobic exercise T2: Combined aerobic and resistance exercise	12	3	60	T1: medium intensity T2: medium intensity	①②③
Yajing Wang 2019 (42)	China	34/31	48.32 ± 7.97/ 46.71 ± 8.10	T1: Intermittent aerobic exercise T2: Continuous aerobic exercise	12	3	50	T1:70%-90%HRmax+Intermittent low intensity; T2:50%-70%HRmax	①②③

T, experimental group; C, control group; M, male; F, female; “—”, not reported; min, minutes; ①, Fasting blood glucose; ②, 2-hour postprandial blood glucose; ③, Glycated hemoglobin; VO2max, maximum oxygen uptake; HRR, reserve heart rate; HRmax, maximum heart rate; 1RM, 1-repetition maximal strength; Borg、RPE, subjective fatigue rating scale.

### 3.3 Quality assessment of included studies

The PEDro scores for the 22 studies ranged from 6 to 8, with an average score of 6.41, suggesting that the overall quality of the included studies was quite high. All 22 studies (21–42) utilized random allocation for participants and adhered to criteria such as baseline similarity, intention-to-treat analysis, inter-group statistical comparisons, point estimates, and measures of variability. Three studies (25, 28, 39) fulfilled the criteria for allocation concealment, one study (21) met the criteria for participant blinding, another study (21) adhered to the criteria for therapist blinding, and six studies (21, 25, 31, 39, 40, 42) complied with the criteria for assessor blinding. Two studies (21, 39) indicated that the dropout rates of participants exceeded 15%. The quality assessment of the included studies can be found in **Table 2**.

**Table 2:** Results of the methodological quality assessment of the included literature.

Studies	Eligibility criteria	Random sequence generation	Allocation concealment	Similar baseline	Blinding of participants	Blinding of therapist	Blinding of outcome assessment	Dropout rates of participants ≤ 15%	Intent-to-treat information	Between group statistical outcome analysis	Point measures and measures of variance	Total Score
Church 2010 (21)	1	1	0	1	1	1	1	0	1	1	1	8
Jorge 2011 (22)	1	1	0	1	0	0	0	1	1	1	1	6
Karstoft 2013 (23)	1	1	0	1	0	0	0	1	1	1	1	6
Oliveira 2012 (24)	1	1	0	1	0	0	0	1	1	1	1	6
Park 2015 (25)	1	1	1	1	0	0	1	1	1	1	1	8
Shenoy 2009 (26)	1	1	0	1	0	0	0	1	1	1	1	6
Tan 2012 (27)	1	1	0	1	0	0	0	1	1	1	1	6
Winding 2018 (28)	1	1	1	1	0	0	0	1	1	1	1	7
Yavari 2012 (29)	1	1	0	1	0	0	0	1	1	1	1	6
Huilan Cheng 2013 (30)	1	1	0	1	0	0	0	1	1	1	1	6
Jiangping Hu 2019 (31)	1	1	0	1	0	0	1	1	1	1	1	7
Tongxin Li 2014 (32)	1	1	0	1	0	0	0	1	1	1	1	6
Yishu Wang 2016 (33)	1	1	0	1	0	0	0	1	1	1	1	6
Yuxin Xu 2019 (34)	1	1	0	1	0	0	0	1	1	1	1	6
Qing Meng 2018 (35)	1	1	0	1	0	0	0	1	1	1	1	6
Qian Liu 2021 (36)	1	1	0	1	0	0	0	1	1	1	1	6
Qi Gu 2021 (37)	1	1	0	1	0	0	0	1	1	1	1	6
Yanping Wu 2022 (38)	1	1	0	1	0	0	0	1	1	1	1	6
Ng 2010 (39)	1	1	1	1	0	0	1	0	1	1	1	7
Terada 2013 (40)	1	1	0	1	0	0	1	1	1	1	1	7
Xiujun Zhao 2019 (41)	1	1	0	1	0	0	0	1	1	1	1	6
Yajing Wang 2019 (42)	1	1	0	1	0	0	1	1	1	1	1	7

### 3.4 The effects of exercise therapy on blood glucose levels in patients with type 2 diabetes

The traditional meta-analysis was limited to studies that utilized a conventional treatment group as the control (21–38), comprising a total of 18 studies. As illustrated in **Table 3**, the fasting blood glucose measure comprised 16 studies (22–24, 26–38) (resulting in a total of 32 analyses). The results of the heterogeneity test showed: *I*²=59%, *P* < 0.0001, with a combined effect size of SMD=-0.58, 95%CI: [-0.77, -0.39], *P* < 0.00001. The postprandial blood glucose measure comprised 13 studies (22–24, 27–33, 36–38), resulting in a total of 26 analyses. The heterogeneity test indicated: *I*²=57%, *P* = 0.0002, with a combined effect size of SMD=-0.51, 95%CI: [-0.73, -0.30], *P* < 0.00001. The glycated hemoglobin measure comprised 18 studies (21–38), resulting in a total of 36 analyses. The heterogeneity test revealed: *I*²=87%, *P* < 0.00001, with a combined effect size of MD=-0.66, 95%CI: [-0.78, -0.54], *P* < 0.00001. High heterogeneity was observed among the studies for all three measures, and a random-effects model was employed for the analysis. These findings suggest that exercise therapy can effectively improve blood glucose levels in patients with type 2 diabetes.

**Table 3:** Meta-Analysis of Blood glucose Indicators.

Indicator	Sample size	Effectiveness[95%CI]	*P*	*I*²/%	*P* ^Heterogeneity^
Fasting blood glucose	639/594	-0.58(-0.77, -0.39)	<0.00001	59	<0.0001
2-hour postprandial blood glucose	459/422	-0.51(-0.73, -0.30)	<0.00001	57	0.0002
Glycated hemoglobin	884/730	-0.66(-0.78, -0.54)	<0.00001	87	<0.00001

In order to investigate the heterogeneity resulting from various exercise regimens, the effect of exercise therapy on blood glucose levels in patients with type 2 diabetes may be influenced by several factors, including exercise type, duration, intensity, frequency, and timing. Since the frequency and duration of exercise were relatively consistent, and some studies provided unclear descriptions of exercise intensity, subgroup analyses were not feasible. Thus, this article performed subgroup analyses specifically examining the effects of exercise type and duration on blood glucose levels. The included studies were categorized based on exercise types into the following groups: intermittent aerobic exercise, continuous aerobic exercise, resistance exercise, and combined aerobic and resistance exercise. In terms of intervention duration, the studies were classified into two categories: 2–12 weeks and 14–18 weeks.

As presented in **Table 4**, subgroup analyses were conducted to investigate potential sources of heterogeneity. For fasting blood glucose, the lowest heterogeneity was identified in the exercise type subgroup (*I*² = 0%) and the intervention duration subgroup (*I*² = 12%), representing a considerable decrease compared to the overall pooled estimate (*I*² = 59%). For 2-hour postprandial blood glucose, both the exercise type and intervention duration subgroups exhibited minimal heterogeneity (*I*² = 0% for both), which markedly contrasted with the overall pooled result (*I*² = 57%). Regarding glycated hemoglobin, the lowest heterogeneity was observed in the exercise type subgroup (*I*² = 9%) and the intervention duration subgroup (*I*² = 48%), again indicating a substantial reduction relative to the overall estimate (*I*² = 87%). These results suggest that both exercise type and intervention duration are probable sources of heterogeneity in the meta-analysis. In comparison to conventional treatment, neither intermittent aerobic exercise nor resistance training demonstrated statistically significant differences in improving postprandial blood glucose levels. Other exercise types and durations were found to be effective in improving blood glucose levels (*P* < 0.01).

**Table 4:** Subgroup analysis of blood glucose in patients with type 2 diabetes mellitus by exercise therapy.

Indicator	Study characteristics	Subgroups	Sample size	MD/SMD	95%CI	*P*	*I*²/%	*P* ^Heterogeneity^
Fasting blood glucose	Exercise form	Intermittent aerobic exercise	48/33	-0.63	-1.08,-0.17	0.007	0%	0.57
		Continuous aerobic exercise	239/224	-0.46	-0.69,-0.23	<0.0001	29%	0.15
		Resistance exercise	122/120	-0.59	-1.01,-0.16	0.007	60%	0.02
		Combined aerobic and resistance exercise	230/217	-0.70	-1.14,-0.26	0.002	78%	<0.0001
	Exercise period	2–12 weeks	455/431	-0.45	-0.67,-0.22	0.0001	62%	<0.0001
		14–48 weeks	184/163	-0.94	-1.19,-0.69	<0.00001	12%	0.33
2-hour postprandial blood glucose	Exercise form	Intermittent aerobic exercise	48/33	-0.58	-1.40,0.24	0.16	64%	0.06
		Continuous aerobic exercise	151/138	-0.42	-0.66,-0.19	0.0004	0%	0.78
		Resistance exercise	112/110	-0.68	-1.44,0.09	0.08	86%	<0.00001
		Combined aerobic and resistance exercise	148/141	-0.47	-0.78,-0.16	0.003	37%	0.15
	Exercise period	2–12 weeks	375/351	-0.41	-0.66,-0.16	0.001	62%	0.0001
		14–48 weeks	84/71	-0.90	-1.24,-0.56	<0.00001	0%	0.73
Glycated hemoglobin	Exercise form	Intermittent aerobic exercise	48/33	-0.40	-0.66,-0.15	0.002	9%	0.33
		Continuous aerobic exercise	311/265	-0.68	-1.01,-0.35	<0.0001	80%	<0.00001
		Resistance exercise	195/161	-0.71	-1.22,-0.20	0.007	77%	<0.0001
		Combined aerobic and resistance exercise	330/271	-0.98	-1.51,-0.45	0.0003	92%	<0.00001
	Exercise period	2–12 weeks	479/444	-0.50	-0.76,-0.25	0.0001	48%	0.006
		14–48 weeks	405/286	-0.69	-0.84,-0.55	<0.00001	95%	<0.00001

### 3.5 Ranking the effectiveness of different exercise therapies on blood glucose levels in patients with type 2 diabetes

#### 3.5.1 Evidence network

**Figure 2:**
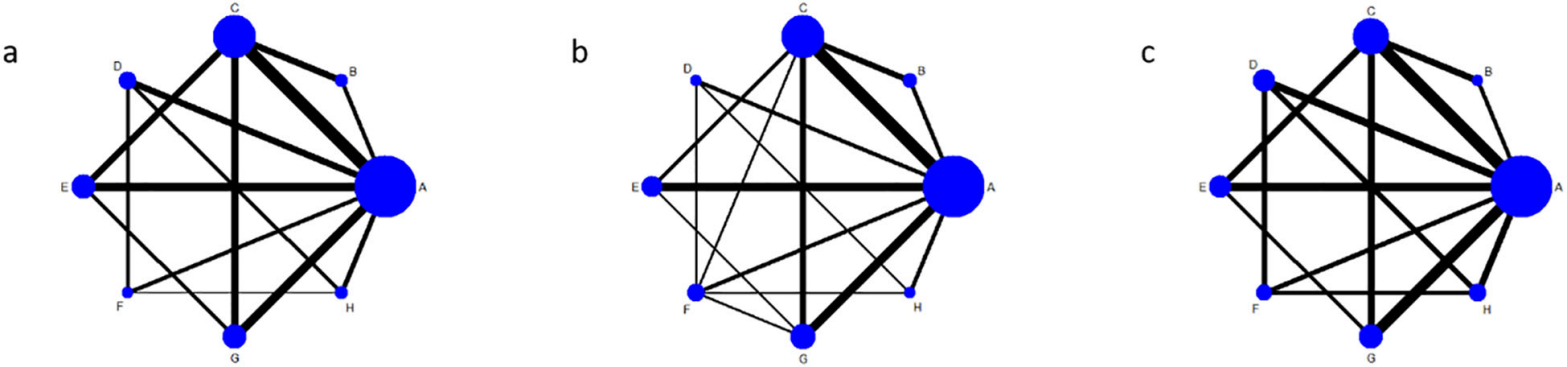
Shows the evidence network diagram of different exercise therapies in the intervention of blood glucose in patients with type 2 diabetes. **(a)**, fasting blood glucose; **(b)**, 2-hour postprandial blood glucose; **(c)**, Glycated hemoglobin; A, conventional treatment; B, Intermittent aerobic exercise for 2 to 12 weeks; C, continuous aerobic exercise for 2 to 12 weeks; D, continuous aerobic exercise for 14 to 48 weeks; E, resistance exercise for 2 to 12 weeks; F, resistance exercise for 14–48 weeks; G, aerobic combined with resistance exercise for 2 to 12 weeks; H, 14–48 weeks of aerobic combined with resistance exercise.

This study explored combinations of exercise components, and subgroup analyses revealed significant variations based on exercise type and duration. Building upon the conventional meta-analysis, a network meta-analysis (NMA) was conducted to evaluate integrated interventions combining exercise type and duration. The NMA incorporated trials that lacked a conventional control group but included multiple exercise intervention arms, resulting in the inclusion of 22 studies. The network of comparisons among interventions is depicted in **Figure 2**. Solid edges between nodes indicate the presence of direct comparative evidence between two interventions. The absence of a connecting line implies that no direct comparisons were available, in which case effects were estimated via indirect evidence. The thickness of each edge is proportional to the amount of available direct evidence. Each node (represented by a circle and corresponding letter) denotes a distinct intervention, and the size of the circle is proportional to the number of participants assigned to that intervention.

The results revealed a relatively complex evidence network among the different exercise interventions. Conventional therapy (A) served as the common reference and was directly compared with most exercise-based interventions. Among these, the most substantial body of direct comparative evidence (indicated by thicker edges in the network graph) was observed for short-term (2–12 weeks) continuous aerobic exercise (C). Furthermore, direct comparisons were also present between long-term (14–48 weeks) combined aerobic and resistance exercise (H) and both long-term continuous aerobic exercise (D) and long-term resistance exercise (F).

#### 3.5.2 Results of network meta-analysis and probability rankings

##### 3.5.2.1 Results and ranking of the network meta-analysis for fasting blood glucose

The analysis of fasting blood glucose included 20 studies (22–24, 26–42), consisting of 11 two-arm studies, 6 three-arm studies, and 3 four-arm studies, encompassing a total of 7 distinct exercise regimens. A random effects model was employed for the analysis, with PSRF values nearing 1.00, suggesting satisfactory convergence and low overall heterogeneity (*I* ^2^ = 0%). Most exercise interventions demonstrated a reduction in fasting blood glucose compared to conventional therapy. The network meta-analysis results demonstrated that, apart from the 2–12 week intermittent aerobic exercise (SMD=-0.766, 95%CI: [-1.734 to 0.168]) and the 2–12 week continuous aerobic exercise (SMD=-0.570, 95%CI: [-1.181 to 0.064]), which exhibited no statistically significant differences when compared to the conventional treatment group, all other exercise therapies significantly lowered fasting blood glucose levels in patients with type 2 diabetes. The most pronounced effect was observed for combined aerobic and resistance exercise with an intervention duration of 14–48 weeks (SMD=-2.106, 95%CI: [-3.042 to -1.176]). The surface under the cumulative ranking curve (SUCRA) values indicated the following probability ranking, in descending order of efficacy: 14–48 weeks of combined aerobic and resistance exercise (0.939), 14–48 weeks of resistance exercise (0.776), 2–12 weeks of resistance exercise (0.660), 2–12 weeks of combined aerobic and resistance exercise (0.518), and 14–48 weeks of continuous aerobic exercise (0.474). For detailed results, please refer to **Table 5**.

**Table 5:** Effects of different exercise programs.

Exercise protocols	Fasting blood glucose		2-hour postprandial blood glucose		Glycated hemoglobin	
	SUCRA value	Effectiveness[95%]	SUCRA value	Effectiveness[95%]	SUCRA value	Effectiveness[95%]
2–12 weeks intermittent aerobic exercise	0.374	-0.766(-1.734, 0.168)	0.561	-1.671(-3.206, 0.013)	0.446	-0.542(-1.293, 0.225)
2–12 weeks continuous aerobic exercise	0.243	-0.570(-1.181, 0.064)	0.439	-1.361(-2.470, -0.216)	0.190	-0.209(-0.732, 0.324)
14–48 weeks continuous aerobic exercise	0.474	-0.993(-1.828, -0.158)	0.483	-1.447(-3.191, 0.113)	0.706	-0.943(-1.557, -0.357)
2–12 weeks resistance exercise	0.660	-1.326(-2.136, -0.434)	0.781	-2.419(-3.530, -0.833)	0.384	-0.462(-1.154, 0.231)
14–48 weeks resistance exercise	0.776	-1.658(-2.899, -0.464)	0.380	-1.113(-3.235, 0.975)	0.682	-0.921(-1.680, -0.191)
2–12 weeks combined aerobic and resistance exercise	0.518	-1.050(-1.671, -0.331)	0.431	-1.331(-2.486, -0.086)	0.589	-0.747(-1.291, -0.195)
14–48 weeks combined aerobic and resistance exercise	0.939	-2.106(-3.042, -1.176)	0.891	-3.177(-5.623, -0.791)	0.947	-1.382(-2.050, -0.747)

##### 3.5.2.2 Results and ranking of the network meta-analysis for postprandial blood glucose at 2 Hours

The analysis of postprandial blood glucose at 2 hours included 15 studies (22–24, 27–33, 36–38, 41, 42), consisting of 9 two-arm studies, 3 three-arm studies, and 3 four-arm studies, encompassing a total of 7 distinct exercise regimens. A random effects model was employed for the analysis, with PSRF values nearing 1.00, suggesting satisfactory convergence and low overall heterogeneity (*I* ^2^ = 0%). The network meta-analysis results demonstrated that, apart from the 2–12 week intermittent aerobic exercise (SMD=-1.671, 95%CI: [-3.206 to 0.013]), the 14–48 week continuous aerobic exercise (SMD=-1.447, 95%CI: [-3.191 to 0.113]), and the 14–48 week resistance exercise (SMD=-1.113, 95%CI: [-3.235 to 0.975]), which exhibited no statistically significant differences compared to the conventional treatment group, all other exercise therapies significantly lowered postprandial blood glucose levels at 2 hours in patients with type 2 diabetes. The most effective intervention was identified as combined aerobic and resistance exercise administered over a period of 14 to 48 weeks (SMD=-3.177, 95%CI: [-5.623 to -0.791]). The surface under the cumulative ranking curve (SUCRA) values indicated the following probability ranking, in descending order of efficacy: 14–48 weeks of combined aerobic and resistance exercise (0.891), 2–12 weeks of resistance exercise (0.781), 2–12 weeks of continuous aerobic exercise (0.439), and 2–12 weeks of combined aerobic and resistance exercise (0.431). For more details, refer to **Table 5**.

##### 3.5.2.3 Results and ranking of the network meta-analysis for glycated hemoglobin

The analysis of glycated hemoglobin included 22 studies (21–42), consisting of 12 two-arm studies, 6 three-arm studies, and 4 four-arm studies, encompassing a total of 7 distinct exercise regimens. A random effects model was employed for the analysis, with PSRF values nearing 1.00, suggesting satisfactory convergence and low overall heterogeneity (*I* ^2^ = 0%). The network meta-analysis results demonstrated that, apart from the 2–12 week intermittent aerobic exercise (MD=-0.542, 95%CI: [-1.293 to 0.225]), the 2–12 week continuous aerobic exercise (MD=-0.209, 95%CI: [-0.732 to 0.324]), and the 2–12 week resistance exercise (MD=-0.462, 95%CI: [-1.154 to 0.231]), which exhibited no statistically significant differences compared to the conventional treatment group, all other exercise therapies significantly lowered glycated hemoglobin levels in patients with type 2 diabetes. The optimal intervention was determined to be combined aerobic and resistance exercise conducted over a period of 14 to 48 weeks (MD=-1.382, 95%CI: [-2.050 to -0.747]). The surface under the cumulative ranking curve (SUCRA) values indicated the following probability ranking, in descending order of efficacy: 14–48 weeks of combined aerobic and resistance exercise (0.947), 14–48 weeks of continuous aerobic exercise (0.706), 14–48 weeks of resistance exercise (0.682), and 2–12 weeks of combined aerobic and resistance exercise (0.589). For more details, refer to **Table 5**.

#### 3.5.3 Publication bias

**Figure 3:**
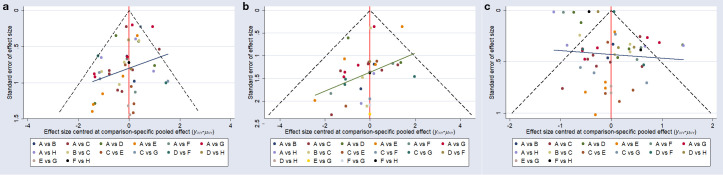
Comparison - corrected funnel plot. **(a)**, fasting blood glucose; **(b)**, postprandial blood glucose at 2 hours; **(c)**, glycated hemoglobin; A, conventional treatment; B, 2–12 weeks of intermittent aerobic exercise; C, 2–12 weeks of continuous aerobic exercise; D, 14–48 weeks of continuous aerobic exercise; E, 2–12 weeks of resistance exercise; F, 14–48 weeks of resistance exercise; G, 2–12 weeks of combined aerobic and resistance exercise; H, 14–48 weeks of combined aerobic and resistance exercise.

A funnel plot was constructed to assess potential publication bias for the glycemic outcomes (**Figure 3**). Most of the included studies were distributed symmetrically around the vertical line (x = 0). The plots for fasting blood glucose (**Figure 3a**), 2-hour postprandial blood glucose (**Figure 3b**), and glycated hemoglobin (**Figure 3c**) each exhibited approximate symmetry, suggesting a low risk of publication bias.

## 4 Discussion

This study demonstrates that exercise therapy significantly improves fasting blood glucose, 2-hour postprandial blood glucose, and glycated hemoglobin (HbA1c) levels in patients with type 2 diabetes mellitus (T2DM). These results are consistent with previous studies (15–17, 43–46). Long-term, regular, and moderate-intensity exercise can elicit therapeutic effects comparable to those achieved with oral hypoglycemic agents. Exercise ameliorates glucose metabolism, enhances insulin secretion, and improves tissue sensitivity to insulin in individuals with T2DM (15, 47). Furthermore, it facilitates the functional recovery of pancreatic β-cells, contributing to improved glycemic control (48). Notably, recent neurophysiological studies have indicated that patients with type 2 diabetes mellitus (T2DM) frequently present with distal neuropathy. This finding underscores the importance of assessing neuropathic status in clinical exercise prescription to enhance intervention safety (49).

This study identified combined aerobic and resistance exercise as the most effective intervention for improving fasting blood glucose, 2-hour postprandial blood glucose, and glycated hemoglobin levels in patients with type 2 diabetes. The glycemic benefits of combined exercise may be attributed to complementary physiological mechanisms. Aerobic exercise contributes to reductions in body fat and enhances the sensitivity and responsiveness of peripheral target tissues to insulin through skeletal muscle activation, thereby promoting glucose utilization and mitigating insulin resistance. Additionally, it improves skeletal muscle perfusion, which facilitates glucose uptake and metabolism (50, 51). Resistance exercise, on the other hand, increases skeletal muscle mass and quality, leading to greater insulin receptor density and sensitivity. The repetitive muscle contractions during resistance training induce localized hypoxia, which stimulates the translocation of glucose transport proteins to the cell surface and enhances glucose uptake efficiency (51, 52). When combined, these exercise modalities synergistically improve muscular strength and maximal oxygen consumption (VO₂max), resulting in sustained enhancements in insulin sensitivity and skeletal muscle glucose disposal. Notably, the glycemic benefits persist longer following combined exercise compared to either modality alone, supporting its superiority in long-term blood glucose management (35). These findings align with recommendations from the American Diabetes Association and the International Diabetes Federation, which endorse regular aerobic and resistance exercise as part of standard T2DM management. Current guidelines advise at least 150 minutes of moderate-intensity aerobic exercise plus two resistance training sessions per week (53, 54).

This study determined that an exercise intervention lasting 14–48 weeks is most effective for improving fasting blood glucose, 2-hour postprandial blood glucose, and glycated hemoglobin (HbA1c) levels. These findings are consistent with previous research by Wu et al., who recommended a 26-week exercise regimen for middle-aged and elderly patients with T2DM to improve HbA1c and fasting glucose (43). Long-term regular exercise enhances skeletal muscle structure and function, improves oxidative capacity, and promotes energy expenditure, thereby reducing overall adiposity and ectopic lipid accumulation in insulin-target tissues such as skeletal muscle and the liver. These adaptations contribute to improved glucose utilization and enhanced insulin secretory function (55). Furthermore, since glycated hemoglobin reflects average blood glucose levels over a period of 2–3 months and is not influenced by short-term glycemic fluctuations, an intervention duration of at least three months is necessary to reliably evaluate exercise-induced improvements in glycemic control.

This study further revealed that a combination of aerobic exercise and resistance training over a period of 14 to 48 weeks yielded the most effective interventions for fasting blood glucose, postprandial blood glucose at 2 hours, and glycated hemoglobin. Fasting blood glucose and glycated hemoglobin levels are essential metrics for assessing patients with type 2 diabetes, owing to their reliability in accurately reflecting both long-term and overall blood glucose levels. In contrast, postprandial blood glucose at 2 hours indicates blood glucose control following food intake, showcasing both sensitivity and dynamism (2). Therefore, a comprehensive assessment incorporating multiple glycemic indicators is essential for devising individualized and effective glucose management strategies. It should be noted that evidence regarding intermittent aerobic exercise programs spanning 14–48 weeks remains limited, resulting in uncertain efficacy outcomes. Further high-quality studies are warranted to clarify its potential benefits.

In recent years, the management strategy for type 2 diabetes mellitus (T2DM) has shifted from a glucocentric approach toward comprehensive management of multiple risk factors, particularly in the context of widely adopted novel pharmacological agents such as SGLT2 inhibitors and GLP-1 receptor agonists (56). Despite the expanding arsenal of glucose-lowering medications and the increasing burden of comorbidities, exercise therapy remains an irreplaceable cornerstone as a fundamental non-pharmacological intervention. Exercise therapy has demonstrated robust efficacy in improving glycemic control in patients with T2DM. Current evidence recommends combined aerobic and resistance exercise administered over 14–48 weeks as the optimal strategy for ameliorating fasting blood glucose, 2-hour postprandial blood glucose, and glycated hemoglobin levels (57–59). This combined exercise recommendation is not only consistent with current clinical guidelines and expert consensus but also provides high-quality, actionable evidence for personalized non-pharmacological management. The prescribed exercise regimen offers several clinical advantages:① High practicality and convenience, owing to minimal requirements for specialized equipment or dedicated venues. ② A favorable safety profile as a non-pharmacological intervention; moderate-intensity exercise is associated with low risk and is suitable for long-term adherence in most patients. ③ Significant therapeutic efficacy, supported by network meta-analyses confirming the regimen’s superiority over conventional therapy across multiple glycemic indicators.

This study has several limitations. First, although random-effects models and Bayesian frameworks were employed, considerable heterogeneity persisted due to variations in exercise type, duration, and intensity across the included trials, which may not have been fully accounted for by subgroup or network analyses. Second, important patient characteristics—such as diabetes duration, comorbidities, and baseline physical function—were not consistently reported in the original studies. In addition, the restriction of the literature search to Chinese and English languages may have resulted in the omission of relevant studies. These factors collectively limit the generalizability of our findings. Furthermore, the current analysis focused exclusively on glycemic outcomes and did not evaluate metrics related to cardiovascular structure or function. Given existing evidence indicating adverse effects of diabetes and hyperglycemia on left ventricular global longitudinal strain (LV-GLS) (60, 61), future research should investigate whether exercise interventions can improve cardiac function parameters—such as LV-GLS assessed via speckle-tracking echocardiography—to provide a more comprehensive evidence base for rehabilitation in T2DM.

In conclusion, this study provides robust evidence that a structured exercise intervention, particularly combining aerobic and resistance training over 14–48 weeks, serves as an effective non-pharmacological strategy for improving key glycemic parameters—including fasting blood glucose, 2-hour postprandial glucose, and HbA1c—in patients with T2DM. The findings reinforce the essential role of exercise therapy within comprehensive diabetes management, highlighting its practicality, safety, and sustained metabolic benefits. While limitations related to heterogeneity and unreported patient variables should be considered, this analysis offers actionable insights for clinical practice and underscores the importance of integrating individualized exercise prescriptions into standard care. Future studies should further explore the long-term effects of exercise on cardiovascular outcomes and functional recovery in diverse T2DM populations.

## Data Availability

The original contributions presented in the study are included in the article/supplementary material. Further inquiries can be directed to the corresponding author.
